# Beneficial effects of antioxidant therapy in crush syndrome in a rodent model: enough evidences to be used in humans?

**DOI:** 10.1186/s13613-018-0431-5

**Published:** 2018-09-27

**Authors:** Patrick M. Honore, David De Bels, Herbert D. Spapen

**Affiliations:** 1 ICU Department, Centre Hospitalier Universitaire Brugmann, 4, Place Arthur Van Gehuchten, 1020 Brussels, Belgium; 2 Universitair Ziekenhuis Brussel, VUB University, Brussels, Belgium

**Keywords:** Astragaloside IV, Antioxidant therapy, Rhabdomyolysis, Crush syndrome, Ischemia–reperfusion injury

Rhabdomyolysis or skeletal muscle breakdown results in a huge release of myoglobin, sarcoplasmic proteins, and electrolytes into the plasma. The term “crush syndrome (CS)” describes muscle destruction after direct trauma, injury, or compression [1]. CS is the archetype of ischemia–reperfusion injury (IRI) which encompasses tissue and cellular damage due to inadequate blood supply followed by resumption of blood flow. Acute kidney injury (AKI) is the most common systemic complication of rhabdomyolysis. It occurs at an incidence ranging between 10 and 55% and is associated with a poor outcome, particularly in the presence of multiple organ failure [1]. Rhabdomyolysis-induced AKI is likely initiated by lack of oxygen, depletion of high-energy molecules, accumulation of toxic metabolites, and intratubular protein precipitation during the ischemia phase but becomes substantially worsened by myoglobin-induced oxidative stress, inflammation, endothelial dysfunction, vasoconstriction, and apoptosis during reperfusion [2]. Despite increasing insight into the pathophysiologic mechanisms underlying CS, treatment has scarcely evolved over time. Akin to any case of CS are adequate surgical management with timely fasciotomies and debridement, ample intravenous fluid resuscitation, mannitol diuresis, urine alkalinization with bicarbonate, serum potassium control, and organ support [1].

The paper by Murata et al. reinvigorates interest in adjuvant antioxidant therapy of CS. The authors studied the effect of adding astragaloside IV (AS-IV) to saline resuscitation on kidney and muscular mitochondrial dysfunction in a rodent model simulating CS [3]. The saponin AS-IV is one of the main constituents of the common herb Huang Qi (Astragalus root) that is frequently used in traditional Chinese medicine. AS-IV affects numerous signaling pathways, has documented anti-inflammatory, antifibrotic, antioxidative, immunoregulatory, and cardioprotective potential, and has been shown to beneficially influence focal cerebral ischemia/reperfusion, cardiovascular and pulmonary disease, liver fibrosis, and diabetic nephropathy [4]. AS-IV also protects against ischemia-induced AKI through targeting inhibition of inflammatory, oxidative stress, and apoptosis pathways [5]. Murata et al. found that a 10-mg/kg AS-IV bolus injection improved hemodynamics, metabolic alterations, AKI, and survival in their model. AS-IV acted as a potent antioxidant on the kidney and as a nitric oxide donor in injured muscle attenuating mitochondrial dysfunction and inflammation [3].

Basic scientists have capitalized on antioxidant actions of different molecules and substances with promising experimental results, as evidenced by improved regional blood flow and oxygen delivery, microcirculatory vasodilation, decreased lactic acidosis, less organ failure, and better survival [6]. However, clinical results of adjuvant antioxidant treatment in critically ill patients were disappointing or inconclusive [7]. This discrepancy between extensive fundamental knowledge and poor clinical application is multifactorial. Knowledge on the intricate role of reactive oxygen species in disease onset and progression of IRI-related pathologies still remains fragmentary. Antioxidant agents also exert pleiotropic effects which may diverge according to temporal administration patterns [7].

The model presented by Murata et al. considerably differs from the clinical situation. IRI-induced oxidative injury in patients depends upon severity and duration of the ischemic insult, concomitant infection or sepsis, and adequacy of resuscitation all of which cannot be correctly estimated in an acute setting. In addition, many patients will be subjected to various and variable forms of cardiovascular (i.e., vasopressor and inotropic drugs), ventilatory, and extracorporeal organ support (e.g., continuous dialysis) which may all influence disease evolution and outcome. Murata et al. also did not assess the impact of standard applied therapeutic interventions in CS. Oxidation of myoglobin is pH dependent, and bicarbonate alkalinization has been shown to reduce tubular cast formation by inhibiting oxidation and precipitation of myoglobin [8]. Apart from acting as an osmotic diuretic facilitating urinary flow, mannitol also prevents myoglobin precipitation and has oxygen radical scavenging capacity [8]. Resuscitation fluids may have significant yet divergent effects on acid–base equilibrium, microcirculation, endothelial cell structure and permeability, and kidney function [9]. For this reason, normal saline may not be the first choice resuscitation fluid in many hospitals!

Some observations and conclusions of the study must be considered with caution. Although the authors claim a dramatic improvement in survival in AS-IV-treated animals, mortality is still 60%. Expression of heme oxygenase-1 which mediates protection against myoglobin toxicity does not markedly differ between animals treated with either saline alone or saline plus AS-IV at 6 and 24 h of reperfusion. The increased mortality associated with significant and unexplained metabolic and respiratory alkalosis in animals that received a 20 mg/kg AS-IV bolus injection is cumbersome. Finally, suggesting a relationship between improved immunosuppression and an excessive inflammatory response following resuscitation with AS-IV is very speculative. Concomitant pro- and anti-inflammatory activity is complex, patient-related, and varies over time. Interpreting or manipulating post-insult immunosuppression definitely requires more insight in metabolic or endocrine pathways, neutrophil and macrophage behavior, epigenetic reprogramming, myeloid-derived suppressor cell activity, and processes of apoptotic and/or autophagic cellular “housekeeping” [10].

Murata et al. produced an exquisite, remarkably well-constructed, and meticulously conducted research effort which opens interesting perspectives for tackling a harsh and particularly lethal disease. It remains to be proven whether these experimental findings add the right notes to the still unfinished symphony of antioxidant treatment in the critically ill.

## Abbreviations

CS: crush syndrome
IRI: ischemia–reperfusion injury
AKI: acute kidney injury
AS-IV: astragaloside IV
